# Qubit Adoption Method of a Quantum Computing-Based Metaheuristics Algorithm for Truss Structures Analysis

**DOI:** 10.3390/biomimetics9010011

**Published:** 2023-12-27

**Authors:** Donwoo Lee, Seungjae Lee, Sudeok Shon

**Affiliations:** School of Industrial Design & Architectural Engineering, Korea University of Technology & Education, 1600 Chungjeol-ro, Byeongcheon-myeon, Cheonan 31253, Republic of Korea; lov1004ely@koreatech.ac.kr (D.L.); leeseung@koreatech.ac.kr (S.L.)

**Keywords:** quantum computing, qubit, meta-heuristics, QbHS algorithm, optimal design

## Abstract

Since the mention of the Fourth Industrial Revolution in 2016, quantum computers and quantum computing (QC) have emerged as key technologies. Many researchers are trying to realize quantum computers and quantum computing. In particular, most of the development and application of metaheuristics algorithms using quantum computing is focused on computer engineering fields. Cases in which the developed algorithm is applied to the optimal design of a building or the optimal design results presented by expanding the algorithm in various directions are very insufficient. Therefore, in this paper, we proposed four methods of adopting qubits to perform pitch adjusting in the optimization process of the QbHS (quantum-based harmony search) algorithm and applied it to TTO (truss topology optimization) using four methods to compare the results. The four methods of adopting qubits have the same or decreased number of qubits adopted as the number of iterations changes. As a result of applying TTO using four methods, convergence performance differed depending on the adoption method, and convergence performance was superior to conventional HS (harmony search) algorithms in all methods. The optimal design of structural engineering using such QC is expected to contribute to the revitalization of future technologies in the architectural field and the field of computer information systems.

## 1. Introduction

Quantum computing (QC) implies solving problems using quantum mechanical characteristics such as entanglement and superposition of qubits [1]. A method that can solve problems with existing computers using QC was first proposed by Feynman in 1982 [2], and it was proven by Deutsch that data processing is possible by applying qubit states [3]. Existing computers and supercomputers require the development of smaller semiconductors to perform more operations, and now it is no longer possible to miniaturize semiconductors. Thus, the development of quantum computers and quantum computing is essential for greater and faster computation [4].

**Figure 1:**
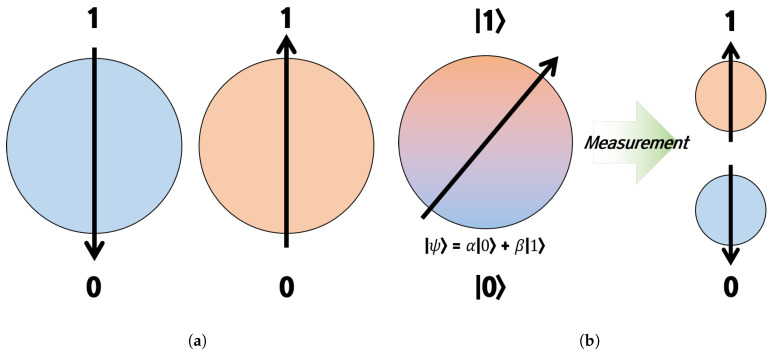
Bit vs. Qubit (**a**) Bit system. (**b**) Qubit system.

The biggest difference between existing computers (supercomputers) and quantum computers is the minimum information processing unit. Existing computers use Bit as the minimum information processing unit, and it is determined and expressed as either zero or one. Quantum computers use qubits as the minimum information processing unit, and qubits are expressed by overlapping zero and one [5]. Figure 1 explains the difference between bit and qubit systems. Both bits and qubits result in zero or one, but qubits have the biggest difference in that they contain probability values for superposition representations [6,7,8]. Quantum computers are characterized by being able to express various information at the same time, so they have the expectation that the operation speed is faster than existing computers (supercomputers) [9].

Metaheuristics algorithms, which are classified into four categories (Evolutionary, Swarm, Physics, and Human behavior) for imitated natural phenomena, are creating new fields that solve optimization problems by combining them with qubit characteristics [10,11,12]. The first case of combination with an evolutionary-based algorithm is the quantum-inspired genetic algorithm (QGA), proposed by Han and Kim in 2000 [13]. Then, in 2002, Han and Kim proposed the quantum-inspired evolutionary algorithm (QEA) [14] and began to create a new field by combining qubit characteristics with metaheuristics algorithms. In 2004, Yang et al. proposed a quantum-inspired particle swarm optimization algorithm (QPSO) in combination with qubit characteristics, the most representative particle swarm algorithm (PSO) in a swarm based algorithm [15]. Among the physics-based algorithms, the harmony search (HS) algorithm was first combined into QC by Layb in 2014 [16], and the quantum-inspired gravitational search algorithm (QIGSA) was proposed by Nezamabadi in 2015 [17]. Among human behavior-based algorithms, teaching–learning-based optimization (TLBO) was combined with QC by Gao et al. in 2019 and was named quantum-inspired teaching–learning-based optimization (QTLBO) [18].

Recently, the amount of research on metaheuristics algorithms has been increasing due to the advantage of being able to solve the NP-hard problem in a short time [19]. In particular, as the number of variables to be processed increases as it begins to be applied to real-life problems, the computational time using existing computers begins to lengthen [20]. Therefore, many studies are being conducted that are creating new fields by combining them with quantum computing with the expectation that design variables can quickly solve many problems. As mentioned above, QC has been combined with various metaheuristics algorithms from the early 2000s to the present to create new fields [21]. However, most metaheuristics algorithms based on QC are only verified for convergence performance through a benchmark function and are mainly applied to binary problems such as switch on/off problems or knapsack problems [16,22,23,24]. Metaheuristics algorithms have solved various engineering problems such as optimal design of architectural structures, robotics, scheduling, and electronic engineering [25,26,27]. Therefore, QC-based metaheuristics algorithms also require efforts to solve various engineering problems, but these attempts are insufficient, especially in the case of applied structural engineering problems. In 2023, Lee et al. performed weight optimization of 20-bar, 24-bar, and 72-bar truss structures with continuous cross-sectional areas using the QbHS (quantum-based harmony search) algorithm [28], and Lee et al. of the same year performed weight optimization of truss structures with discrete cross-sectional areas using the QbHS algorithm [29]. In line with the development of metaheuristics algorithms using QC, it needs to be applied to optimization problems in various architectural engineering fields, and the development and improvement of metaheuristics algorithms using QC to solve architectural engineering problems is required.

Therefore, in this paper, we intend to perform the optimal design of the truss structure using the QbHS algorithm that combines QC and HS algorithms. In particular, we propose four methods in which qubits are adopted in the process of the QbHS algorithm, and we compare the results derived when each of the four methods is applied to truss topology optimization (TTO). Section 2 describes the structure of the QbHS algorithm, and Section 3 describes four methods in which qubits are adopted. Section 4 defines TTO problems and compares and analyzes TTO results using the four methods. Finally, Section 5 concludes this paper.

## 2. QbHS Algorithm

**Figure 2:**
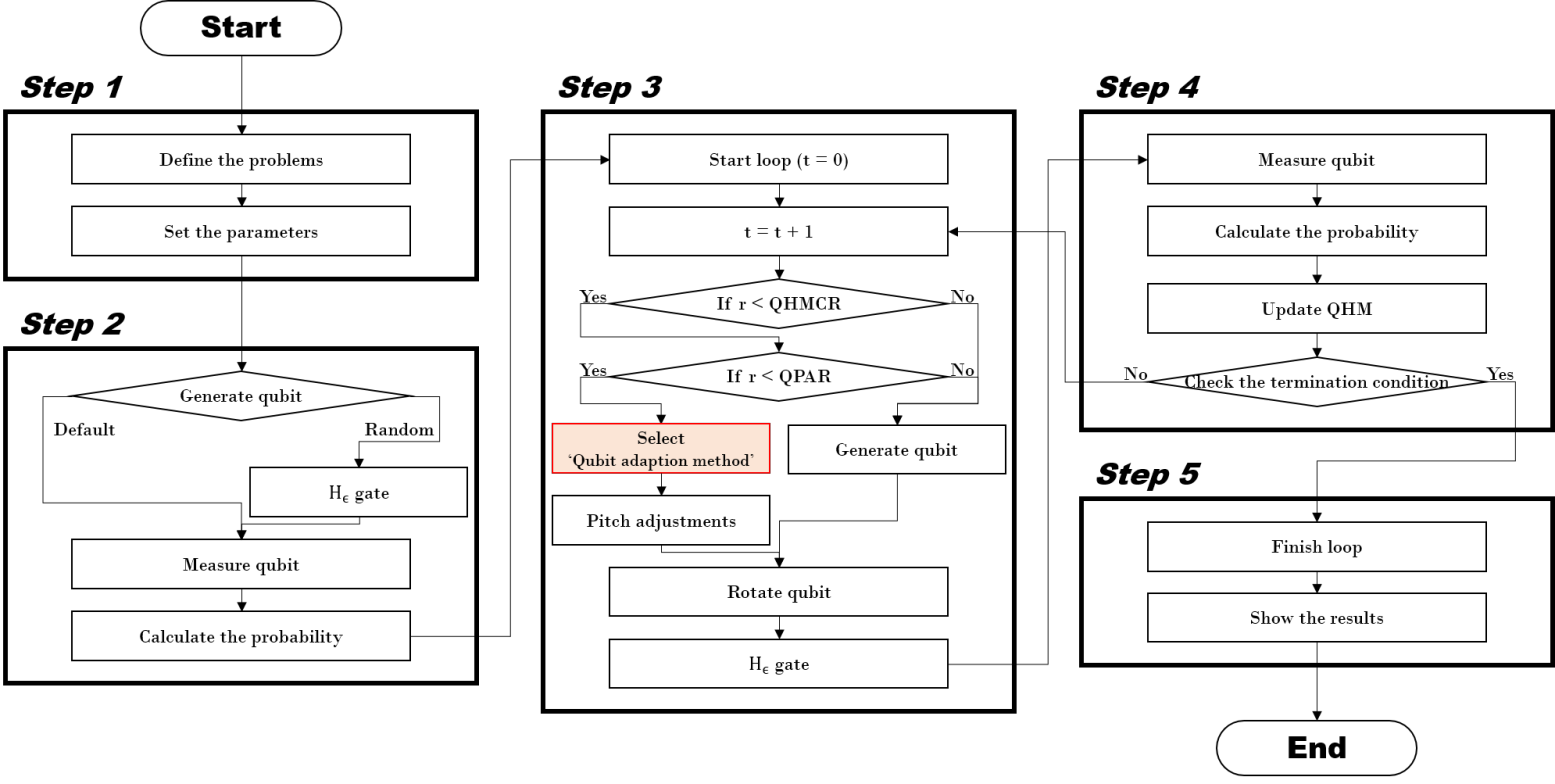
Flowchart of the QbHS algorithm.

The QbHS algorithm combines QC and HS algorithms, and it was proposed by Lee et al. to solve the real problem [28]. The QbHS algorithm consists of five steps, like the HS algorithm proposed by Geem et al. in 2001 [30]. Figure 2 is a flowchart of the QbHS algorithm, implemented using Matlab.

In Step 1, the problem of the optimization target is defined and the parameters used in the algorithm are defined.

In Step 2, Equation (1) is constructed by creating a QHM (quantum harmony memory) and initializing the initial state of the qubit. Here, *N* is the dimension of the problem, and QHMS (quantum harmony memory size) is the size of the QHM .

(1) $$\begin{array}{c} QHM=\left[\begin{array}{ccc} x_{1}^{1} & \cdots & x_{N}^{1}\\ \vdots & \vdots & \vdots \\ x_{1}^{QHMS} & \cdots & x_{N}^{QHMS} \end{array}\right] \end{array}.$$

The design variables that constitute HM (harmony memory) of the HS algorithm are composed of decimal variables. However, the design variables of QHM represent the measured values of qubits in binary. In other words, each design variable can be defined as Equation (2), and *m* means the number of qubits. Each $q_{m}$ contains zero or one information, such as in Equation (3), and is expressed as zero or one through the measurement of qubits. ${\left|\alpha \right|}^{2}$ and ${\left|\beta \right|}^{2}$ mean the probability that zero or one is chosen and must always satisfy Equation (4). Also, Lee et al. classified two algorithms according to the qubit initialization method, and in this paper, the initial probability of zero and one is initialized to 50% [28].

(2) $$x_{j}^{i}=\left\{q_{1},q_{2},...,q_{m}\right\},$$

(3) $$q_{m}=\left[\begin{array}{c} \alpha_{m}\\ \beta_{m} \end{array}\right],$$

(4) $$|\alpha_{i}{|}^{2}+{\left|\beta_{i}\right|}^{2}=1.$$

In Step 3, if *r* (random variable from zero to one) has a value smaller than QHMCR (quantum harmony memory considering rate) and QPAR (quantum pitch adjusting rate), pitch adjusting is performed by adopting a qubit. Pitch adjusting is performed using the amplitude of the qubit ( |α| ) adopted in the current iteration (*t*) and can be expressed as Equations (5) and (6).

(5) $$\left\{\begin{array}{cc} \alpha^{t+1}={\left|\alpha^{t}\right|}^{2}+r\times Qbw & r<0.5\\ \alpha^{t+1}={\left|\alpha^{t}\right|}^{2}-r\times Qbw & else \end{array}\right.,$$

(6) $$Qbw=0.7\times \left(0.9\times qbw_{max}\times \exp\left(\frac{log\left(qbw_{min}/qbw_{max}\right)}{0.7}\right)\times \frac{t}{t_{max}}\right).$$

Lee et al. proposed using the probability average of qubits as a method of adopting qubits for pitch adjusting [28,29]. However, in this paper, we propose a new qubit adoption method using a new equation and compare the results derived by applying each adoption method to the optimal design of the truss structure.

The qubit on which the pitch adjusting is performed rotates using Equation (7) and accumulates information. θ is determined by Table 1, Equations (8) and (9).

(7) $$\left\{\begin{array}{c} \alpha^{t+1}\\ \beta^{t+1} \end{array}\right\}=\left[\begin{array}{cc} \cos(\theta ) & -\sin(\theta )\\ \sin(\theta ) & \cos(\theta ) \end{array}\right]\left\{\begin{array}{c} \alpha^{t}\\ \beta^{t} \end{array}\right\}.$$

(8) $$\theta_{P}=\theta_{r}\times \pi ,$$

(9) $$\theta =\Delta \theta \times sign(\alpha_{i}\beta_{i}).$$

**Figure 3:**
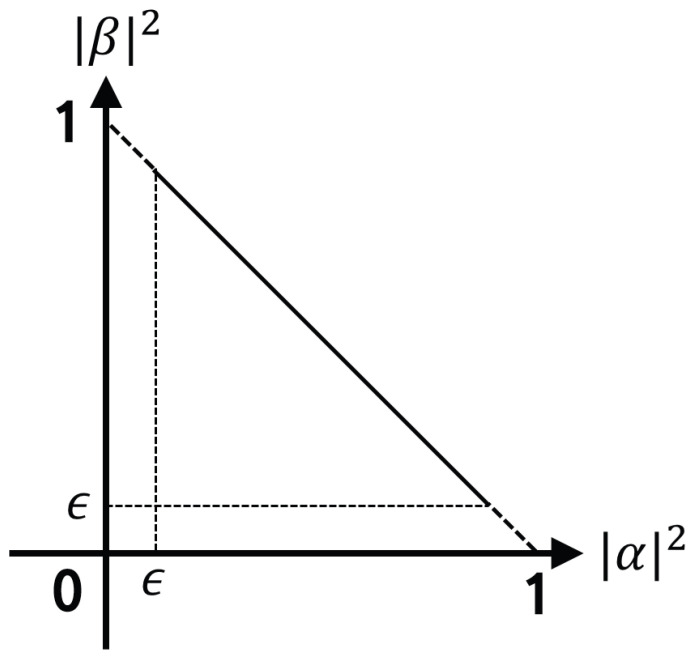
Concept of the $H_{\epsilon }$ gate.

The rotated qubit passes through the $H_{\epsilon }$ gate. It is difficult to escape by itself when the probability of qubit falls into local minima with full convergence to zero or one. Therefore, the $H_{\epsilon }$ gate is used for the purpose of preventing complete convergence of qubits using the initially determined values [31]. Figure 3 is the concept of the $H_{\epsilon }$ gate.

In Step 4, the qubits of the existing QHM and QHM changed by Step 3 are measured to determine whether to update. Using the measured result, the qubit passes through the rotation gate, and the probability information of the qubit is updated again. Qubit probability information is calculated by Equations (10) and (11). Therefore, the probability information of qubits converges to zero or one as the number of iterations increases.

(10) $$C_{av}=\left(\frac{1}{n}\sum_{j=1}^{n}C_{b}\left(q_{j}\right)\right),$$

(11) $$C_{b}\left(q\right)=\frac{1}{m}\sum_{i=1}^{m}\left|1-2\right|\alpha_{i}{|}^{2}|\;\mathrm{or}\;C_{b}\left(q\right)=\frac{1}{m}\sum_{i=1}^{m}\left|1-2\right|\beta_{i}{|}^{2}|.$$

In Step 5, if the termination condition is met, it is terminated. If the termination condition is not met, return to Step 3 and realized and it is repeated until the termination condition is met.

## 3. Qubit Adaption Method

### 3.1. Method I

**Figure 4:**
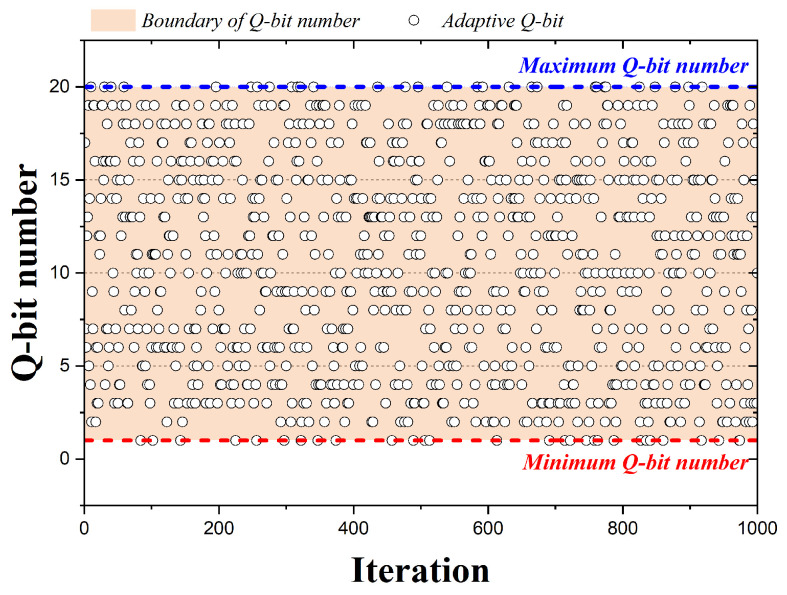
Adaption Method I according to iteration.

In the case of HS algorithms, pitch adjusting is performed by adopting one of the design variables, but QbHS algorithms perform pitch adjusting by adopting one of the qubits constituting the design variable. Figure 4 is a graph representing the qubits adopted by the 1000 iterations when the QbHS algorithm uses Method I. The pink area is the total number of qubits initially set and is assumed to be 20 qubits. In the graph, circles represent qubits adopted for each iteration number, and blue or red lines are the maximum or minimum number of qubits that can be adopted. If the QbHS algorithm uses Method I, there is a total of 20 qubits that can be adopted in each iteration, and they have the same range in all iterations. Therefore, using Method I, the number of qubits that can be pitch adjusted in all iterations does not change, and no other parameters are required except for the number of qubits initially set.

### 3.2. Method II

**Figure 5:**
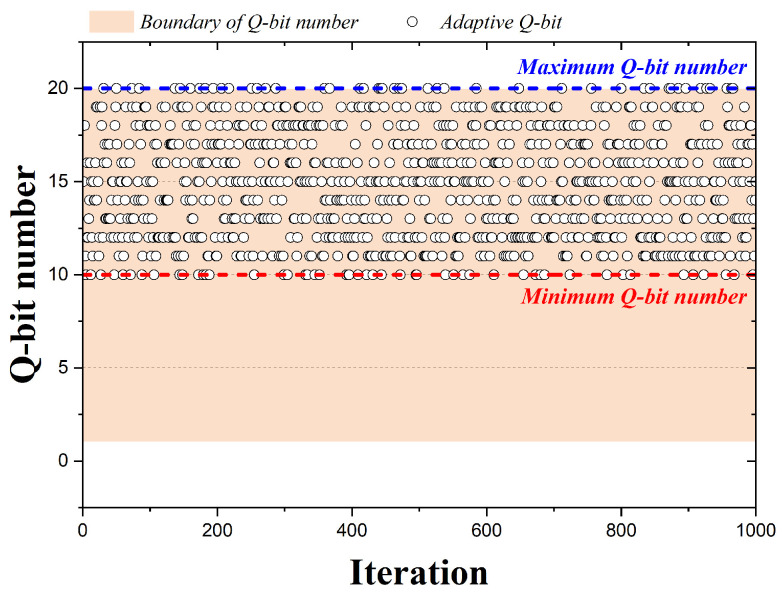
Adaption Method II according to iteration ( BWQ=0.5 ).

Figure 5 is a graph representing the qubits adopted by the 1000 iterations when the QbHS algorithm uses Method II. As shown in Figure 4, the total number of qubits is the same, but the number of qubits adopted for pitch adjusting is from 10 to 20. The minimum number of qubits that can be adopted is determined as Equation (12). Here, $Qubit_{T}$ is the total number of qubits initially determined, and BWQ is a value determined initially from zero to one. Depending on the size of BWQ the maximum and minimum number of qubits that can be adopted for pitch adjusting vary. If BWQ has a size of 0.5, the minimum number of qubit is 10, expressed as Figure 5. Therefore, Method II is similar to Method I, but BWQ can be used to adjust the minimum number of qubits performing pitch adjusting.

(12) $$Qubit_{min}=round\left(Qubit_{T}\times \left(1-BWQ\right)\right)$$

### 3.3. Method III

**Figure 6:**
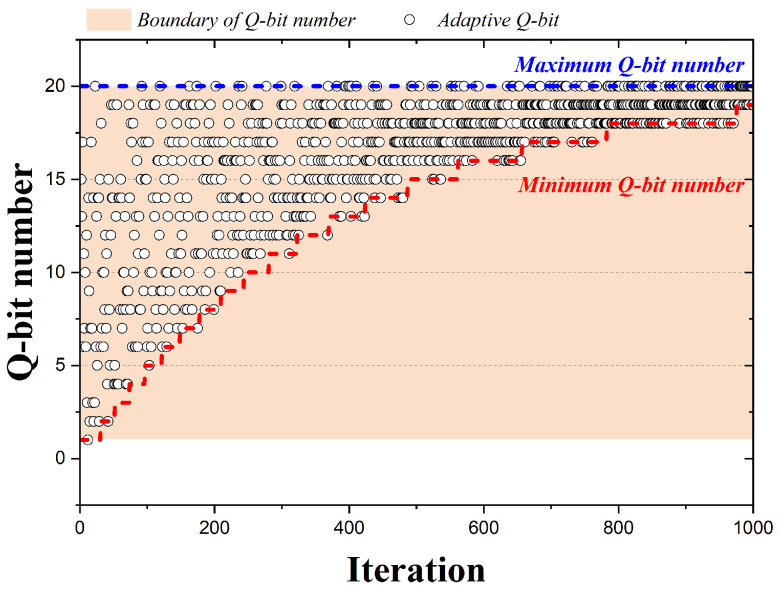
Adaption Method III according to iteration.

Figure 6 is a graph representing the qubits adopted by the 1000 iterations when the QbHS algorithm uses Method III. A total of 20 qubits are assumed, and the range of qubits that can be adopted for pitch adjusting decreases nonlinearly as the number of iterations progresses. The minimum number of qubits that can be adopted changes using Equation (13), and τ is calculated by Equation (14). Here, $BWQ_{max}$ and $BWQ_{min}$ have values from zero to one and are initially determined. *t* is the current number of iterations, $t_{max}$ is the maximum number of iterations. If $BWQ_{max}$ and $BWQ_{min}$ have 1.0 and 0.07, the adoption of qubits that change with iteration change is expressed in Figure 6. Therefore, in Method III, as the number of iterations progresses, the qubits that may be adopted decrease nonlinearly.

(13) $$Qubit_{min}=round\left(Qubit_{T}\times \left(1-\tau \right)\right),$$

(14) $$\tau =BWQ_{max}\times \exp\left(\log\left(\frac{BWQ_{min}}{BWQ_{max}}\right)\times \frac{t}{t_{max}}\right).$$

### 3.4. Method IV

**Figure 7:**
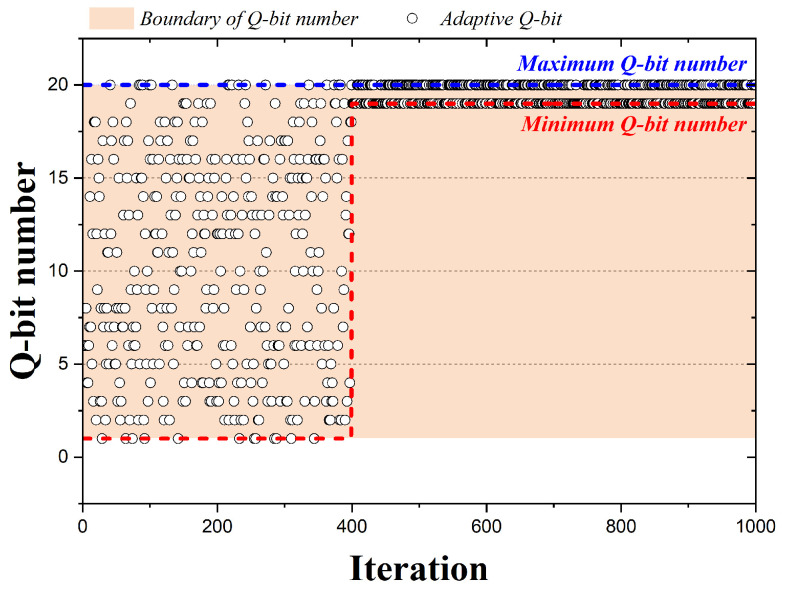
Adaption Method IV according to iteration.

Figure 7 is a graph representing the qubits adopted by the 1000 iterations when the QbHS algorithm uses Method IV. A total of 20 qubits are assumed, and the range of qubits that can be adopted for pitch adjusting decreases sharply in the number of arbitrary iterations. Method IV uses the probability average of qubits. The probability average of qubits is calculated by Equation (10) and can be defined as Equation (15). Here, tolBW is the initially set probability average. The probability average of qubits according to the number of iterations was arbitrarily set, and the qubits that can be adopted when tolBW and $BWQ_{min}$ have values of 0.95 and 0.07 are shown in Figure 7. Therefore, Method IV adopts one of all qubits, as in Method I, when the probability average of qubits is less than tolBW . In other cases, the number of qubits that can be adopted as in Method II is reduced.

(15) $$Qubit_{min}=\left\{\begin{array}{cc} 1 & if\;\;C_{av}<tolBW\\ round(Qubit_{T}\times \left(1-BWQ_{min}\right)) & else \end{array}\right..$$

## 4. Numerical Example

**Figure 8:**
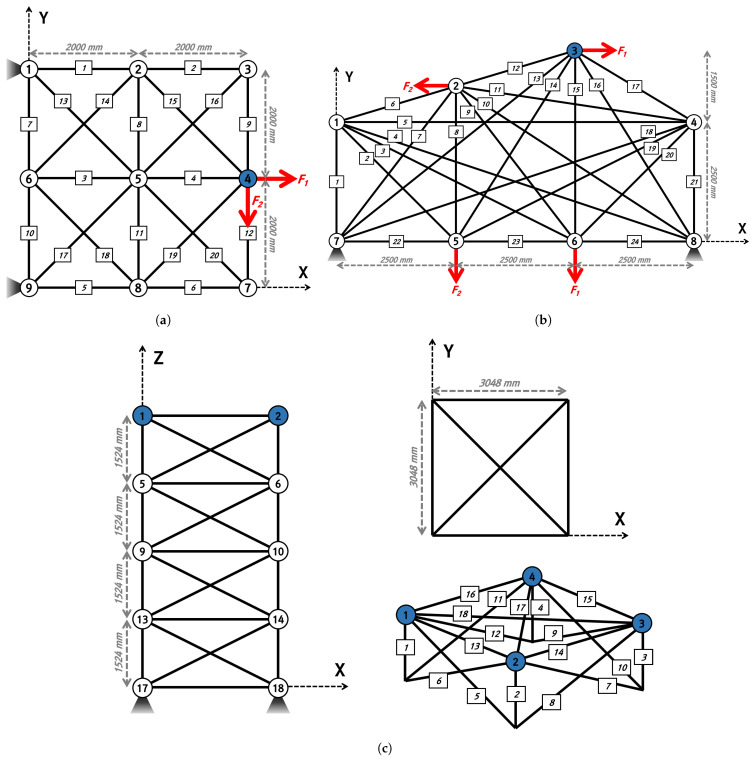
Numerical examples for truss topology optimization: (**a**) 20-bar; (**b**) 24-bar; (**c**) 72-bar.

The effects of the four methods for the adoption of qubits on the convergence performance of the TTO problem were analyzed. The truss structure is a structure in which the member acts only on compression and tensile forces and is an efficient structure that can make long spans without pillars inside. Due to these characteristics, it is widely applied to space trusses, domes, bridges, etc. Therefore, this paper intends to compare the convergence performance according to the qubit adoption method using a simple truss structure example. The TTO problem used 20-bar, 24-bar, and 72-bar truss structures, and the initial shape of the structure is shown in Figure 8. In addition, mass is lumped into the blue nodes of each truss structure.

The mathematical modeling used to solve TTO problems using the QbHS algorithm is the same as in Equation (16). Equation (16) aims to minimize the weight of the truss. Here, ρ means the density of the member, $B_{i}$ means the topology variable, *A* means the cross-sectional area of member, *L* means the length of the member, and *n* means the number of the member. A total of seven constraints are used, ranging from $G_{1}$ to $G_{7}$ and a penalty is imposed if they do not meet the constraints [32,33].

(16) $$\begin{array}{cc} \mathrm{Minimize}\; & Weight\left(x\right)=\rho \sum_{i=1}^{n}B_{i}A_{i}L_{i}, \end{array}$$

(17) $$\begin{array}{cc} \mathrm{Subject}\;\mathrm{to}\; & G_{k}\left(x\right)\leq 0,\;k=1,2,3,4,5,6,7,\\ \; & \\ \; & G_{1}=\left|B_{i}\sigma_{i}\right|-\sigma_{i}^{max}\leq 0\\ \; & G_{2}=\left|\delta \right|-\delta^{max}\leq 0\\ \; & G_{3}=\left|B_{i}\sigma_{i}^{c}\right|-\sigma_{i}^{cr}\leq 0\\ \; & G_{4}=f_{r}-f_{r}^{max}\leq 0\\ \; & G_{5}=A_{min}\leq A_{i}\leq A_{max}\\ \; & G_{6}=Check\;\;validity\;\;of\;\;structure\\ \; & G_{7}=Check\;\;kinematic\;\;stability \end{array}.$$

Table 2 is a parameter of the QbHS algorithm used to solve TTO problems in 20-bar, 24-bar, and 72-bar truss structures. The convergence performance according to the four methods of the QbHS algorithm and the convergence performance of the existing HS algorithm were also compared. Direct comparison between quantum computing-based metaheuristics algorithms and conventional decimal-based metaheuristics algorithms is difficult because the computational methods are different inside. However, the convergence performance of the algorithm was simply compared with the same number of iterations and parameters for the interpretation of existing HS algorithms were also included in Table 2. In addition, each interpretation was repeated 50 times.

### 4.1. The 20-Bar Truss Structure

The initial shape of the 20-bar truss structure is shown in Figure 8a. The 20-bar truss structure consists of nine nodes and 20 members, with a design variable ( $x_{i}$ ) of 20. In addition, the physical properties of the structure are in Table 3. As for constraints, the stress of the member, displacement of Node 4, and natural frequencies are 172.43 MPa, 10 mm, $f_{1}\geq 60$ Hz, and $f_{2}\geq 100$ Hz.

**Figure 9:**
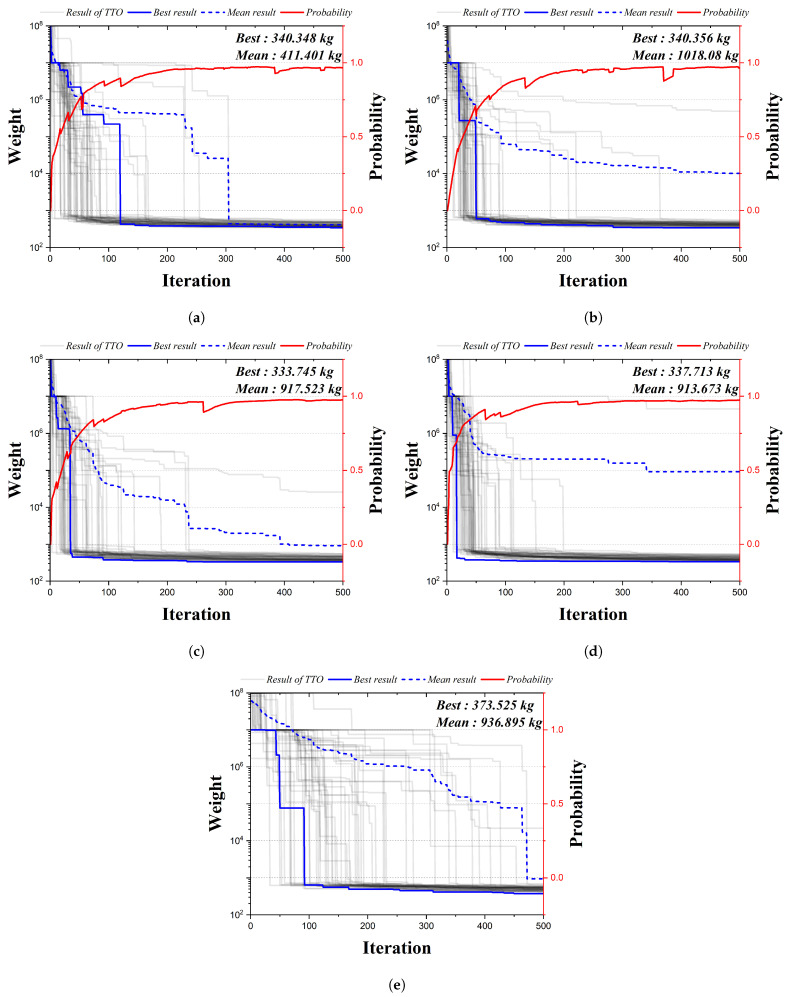
Convergence graph of 20-bar TTO results: (**a**) Method I; (**b**) Method II; (**c**) Method III; (**d**) Method IV; (**e**) HSA.

Figure 9 is the convergence graph of the 20-bar truss structure using the existing HS algorithm and the four qubit adoption methods of the QbHS algorithm. The solid gray line is interpreted 50 times each, the solid blue line is the best weight, the dotted blue line is the mean weight, and the solid red line is the probability of the qubit. Since the existing HS algorithms do not perform operations using qubits, probability is not expressed. The results of the four qubit adoption methods of the QbHS algorithm and the results of the existing HS algorithm all converge to one value, and the probability of qubit also converges to a value close to one. As the minimum weight and mean weight, Method I derives 340.348 kg and 411.401 kg, and Method II derives 340.356 kg and 1018.08 kg. Method III derives 333.745 kg and 917.523 kg, and Method IV derives 337.713 kg and 913.673 kg. When comparing the minimum weight, it is confirmed that the convergence performance is the best when using Method III and that there is a difference in convergence performance for each method. In addition, the existing HS algorithm derives a value of 373.525 kg but increases by up to 11.9% over the results of the QbHS algorithm.

Table 4 is the size of the cross-sectional area of each member derived from the analysis result, and the blank means that there is no element. Method I, Method III, and Method IV adopt a total of 8 elements, and Method II adopts a total of 10 elements. A total of 11 elements of HSA are adopted. Similar topologies are derived from all results except those from HSA. In addition, although there is a difference in the size of the cross-sectional area, it can be confirmed that all of the cross-sectional constraints are satisfied.

### 4.2. The 24-Bar Truss Structure

The initial shape of the 24-bar truss structure is shown in Figure 8b. The 24-bar truss structure consists of eight nodes and 24 members, with a design variable ( $x_{i}$ ) of 24. In addition, the physical properties of the structure are expressed in Table 5. As for constraints, the stress of the member, displacement of Nodes 5 or 6, and natural frequency are 172.43 MPa, 10 mm, and $f_{1}\geq 30$ Hz.

**Figure 10:**
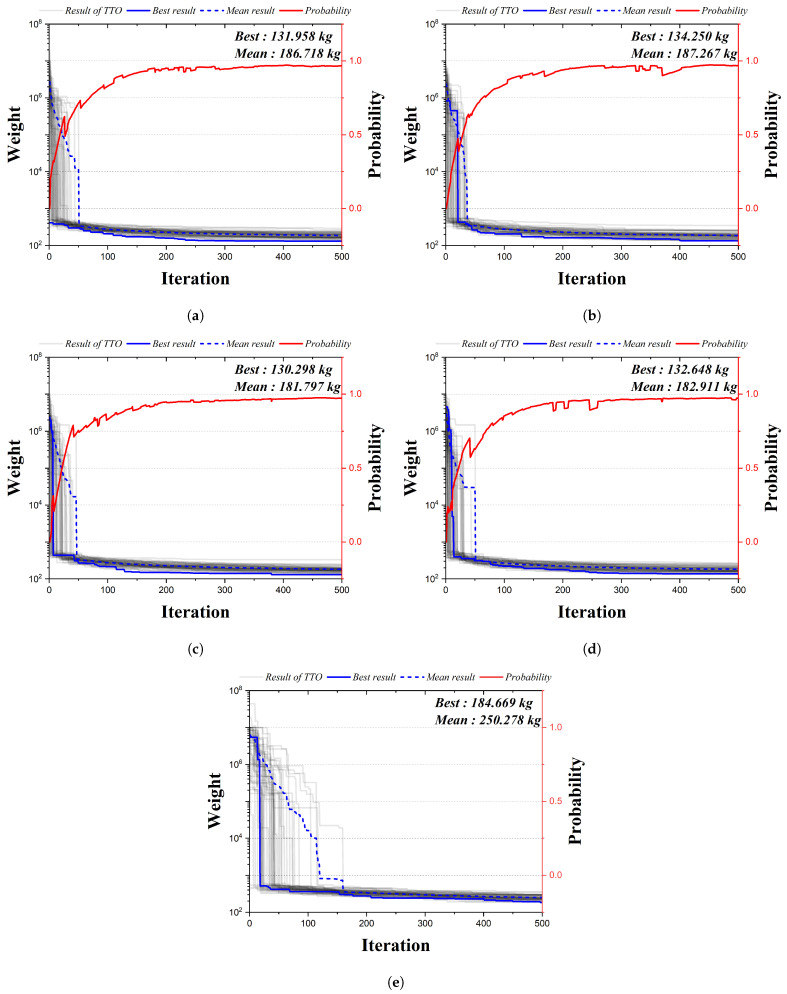
Convergence graph of 24-bar TTO results: (**a**) Method I; (**b**) Method II; (**c**) Method III; (**d**) Method IV; (**e**) HSA.

Figure 10 is the convergence graph of the 24-bar truss structure using the existing HS algorithm and the four qubit adoption methods of the QbHS algorithm. The results of the four qubit adoption methods of the QbHS algorithm and the results of the existing HS algorithm all converge to one value, and the probability of qubit also converges to a value close to one. As for the minimum weight and mean weight, Method I derives 131.958 kg and 186.718 kg, and Method II derives 134.250 kg and 187.267 kg. Method III derives 130.298 kg and 181.797 kg, and Method IV derives 132.648 kg and 182.911 kg. When comparing the minimum weight, it is confirmed that the convergence performance is the best when using Method III and that there is a difference in convergence performance for each method. In addition, the existing HS algorithm derives a value of 184.669 kg but increass by up to 41.7% over the results of the QbHS algorithm.

Table 6 presents the size of the cross-sectional area of each member derived from the analysis result. A total of 8 members are adopted in all methods, and HSA adopts a total of 10 members. Method II and Method III have the same topology derived, but the results of Method I, Method IV, and HSA have different topology-derived structures. Finally, there is a difference in the size of the cross-sectional area, but it can be confirmed that all of the cross-sectional constraints are satisfied.

### 4.3. The 72-Bar Truss Structure

The initial shape of the 72-bar truss structure is shown in Figure 8c. The 72-bar truss structure consists of 20 nodes and 72 members, with a design variable ( $x_{i}$ ) of 16. In addition, the physical properties of the structure are presented in Table 7. As for constraints, the stress of the member, displacement of Nodes 1, 2, 3, 4, and natural frequencies are 172.375 MPa, 6.35 mm, $f_{1}\geq 4$ Hz, and $f_{3}\geq 6$ Hz.

**Figure 11:**
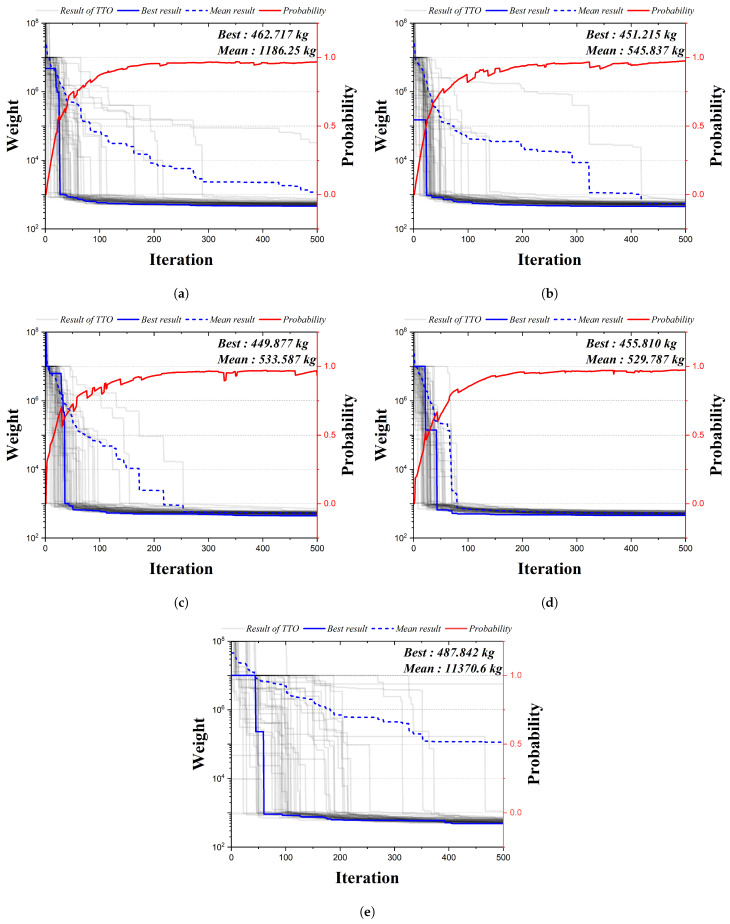
Convergence graph of 72-bar TTO results: (**a**) Method I; (**b**) Method II; (**c**) Method III; (**d**) Method IV; (**e**) HSA.

Figure 11 is the convergence graph of the 72-bar truss structure using the existing HS algorithm and the four qubit adoption methods of the QbHS algorithm. The results of the four qubit adoption methods of the QbHS algorithm and the results of the existing HS algorithm all converge to one value, and the probability of qubit also converges to a value close to one. As the minimum and average weight, Method I derives 462.717 kg and 1186.25 kg, and Method II derives 451.215 kg and 545.837 kg. Method III derives 449.877 kg and 533.587 kg, and Method IV derives 455.810 kg and 529.787 kg. When comparing the minimum weight, it is confirmed that the convergence performance is the best when using Method III and that there is a difference in convergence performance for each method. In addition, the existing HS algorithm derives a value of 487.842 kg, but increases by up to 8.4% over the results of the QbHS algorithm.

Table 8 presents the size of the cross-sectional area of each member derived from the analysis result. In Method II and Method III, a total of 9 members are adopted to derive the same topology, and Method I, Method IV, and HSA adopt a total of 10 members. Although there is a difference in the size of the cross-sectional area, it can be confirmed that all of the cross-sectional constraints are satisfied.

## 5. Conclusions

Recently, as QC has begun to become an issue, many studies have been conducted on the combining or application of QC in many ways. In this paper, an analysis was performed using the QbHS algorithm that combines the HS algorithm, one of the QC and metaheuristics algorithms. In Step 3, the most important step in the QbHS algorithm, four methods of adopting qubits for pitch adjusting were proposed and compared to the results of the TTO problem.

Four methods were proposed so that the number of qubits adopted is the same or changes according to the change in the number of iterations. Method I consists in the number of qubits adopted being the same for all iterations, and Method II is adopted as much as the initially determined variable (BWQ) in the total number of qubits. Method III nonlinearly decreases the number of qubits that can be adopted for pitch adjusting as the number of iterations progresses, and Method IV uses the probability average of qubits to reduce the number of qubits that can be adopted when the probability average of qubits becomes an initially determined variable (tolBW).

In addition, the results of the qubit adoption method were compared by applying it to the TTO problem and compared with the results of the existing HS algorithm. Convergence performance was different according to the adoption method, and adoption Method 3 derived values of 333.745 kg, 130.298 kg, and 449.877 kg, showing the best convergence performance in all TTO examples. In addition, although it is difficult to accurately compare with the existing HS algorithm using decimal variables, it showed better results than the results of the existing HS algorithm. This characteristic is believed to be due to the fact that the exploitation performance has a greater impact on the convergence performance than the exploration performance as the number of later iterations increases.

QC has become an issue, and many researchers are conducting research, but cases of solving problems in structural engineering still need improvement. It is necessary to approach and solve problems in various structural engineering fields using QC for new access to future technologies and convergence of technologies from various perspectives. It is necessary to develop a quantum computing-based algorithm that can easily be applied to architectural engineering and quickly converge. In addition, this paper compared and analyzed the results using the Truss example, but it needs to be applied to optimize a long-span dome structure with many design variables and a vast search space analyzed.

## Figures and Tables

**Table 1:** Look-up table for qubit rotation.

$x_{i}$	$b_{i}$	f(x)<f(b)	Δθ	sign ( $\alpha_{i}\beta_{i}$ )			
				$\alpha_{i}\beta_{i}>0$	$\alpha_{i}\beta_{i}<0$	$\alpha_{i}=0$	$\beta_{i}=0$
0	0	True	0	0	0	0	0
0	0	False	0	0	0	0	0
0	1	True	$\theta_{P}$	1	−1	0	±1
0	1	False	0	0	0	0	0
1	0	True	$-\theta_{P}$	1	−1	±1	0
1	0	False	0	0	0	0	0
1	1	True	0	0	0	0	0
1	1	False	0	0	0	0	0

**Table 2:** Parameters for TTO.

Algorithm	Parameters
QbHSA	QHMS = 10, Mea. = 2, QHMCR = 0.9, QPAR = 0.1, Qubit = 20, ϵ = 0.01, $\theta_{r}$ = 0.06, ${\mathrm{qbw}}_{max}$ = 1.0, ${\mathrm{qbw}}_{min}$ = 0.1, BWQ = 0.3, ${\mathrm{BWQ}}_{max}$ = 1.0, ${\mathrm{BWQ}}_{min}$ = 0.01, tolBW = 0.95
HSA	HMS = 10, HMCR = 0.9, PAR = 0.1, bw = 0.03

**Table 3:** Conditions of 20-bar truss structures.

Lumped Mass	*E*	ρ	*A*	Load	
				Case 1	Case 2
200 kg	69,000 MPa	2740 kg/ $m^{3}$	−100 ${\mathrm{cm}}^{2}$ ≤ x ≤ 100 ${\mathrm{cm}}^{2}$	$F_{1}$ = 500 kN, $F_{2}$ = 0 kN	$F_{1}$ = 0 kN, $F_{2}$ = 500 kN

**Table 4:** The 20-bar TTO results (Unit: ${\mathrm{cm}}^{2}$ ).

Design Variable	Method I	Method II	Method III	Method IV	HSA
A1	31.259	50.001	51.575	68.115	39.252
A2	-	-	-	-	-
A3	-	-	-	-	-
A4	-	-	-	-	5.017
A5	75.043	33.328	51.319	39.845	77.294
A6	-	-	-	-	13.740
A7	-	-	-	-	-
A8	31.630	53.155	52.735	66.725	54.288
A9	-	-	-	-	-
A10	-	4.212	-	-	-
A11	53.137	65.924	33.804	42.621	90.668
A12	-	-	-	-	2.100
A13	49.327	56.260	68.946	81.251	56.214
A14	-	-	-	-	-
A15	53.520	83.315	63.546	59.729	55.469
A16	-	-	-	-	-
A17	-	3.259	-	-	-
A18	87.500	51.661	50.025	42.311	72.267
A19	-	-	-	-	-
A20	75.002	53.415	75.468	60.104	53.204

**Table 5:** Conditions of 24-bar truss structures.

Lumped Mass	*E*	ρ	*A*	Load	
				Case 1	Case 2
500 kg	69,000 MPa	2740 kg/ $m^{3}$	−40 ${\mathrm{cm}}^{2}$ ≤ x ≤ 40 ${\mathrm{cm}}^{2}$	$F_{1}$ = 50 kN, $F_{2}$ = 0 kN	$F_{1}$ = 0 kN, $F_{2}$ = 50 kN

**Table 6:** The 24-bar TTO results (Unit: ${\mathrm{cm}}^{2}$ ).

Design Variable	Method I	Method II	Method III	Method IV	HSA
A1	-	-	-	-	-
A2	-	-	-	-	4.938
A3	-	-	-	-	5.383
A4	-	-	-	-	-
A5	-	-	-	-	-
A6	-	-	-	-	14.560
A7	20.039	22.523	20.625	20.080	26.305
A8	5.025	3.145	4.064	5.078	10.875
A9	-	-	-	-	1.402
A10	-	0.113	0.060	-	-
A11	-	-	-	-	-
A12	0.236	-	-	0.0001	-
A13	20.001	20.642	20.081	20.002	20.380
A14	-	-	-	-	-
A15	5.000	5.159	3.798	5.522	10.767
A16	25.001	24.064	24.071	25.020	24.550
A17	-	-	-	-	-
A18	-	-	-	-	-
A19	-	-	-	-	-
A20	-	-	-	-	-
A21	-	-	-	-	-
A22	-	1.250	0.636	0.198	-
A23	0.626	-	-	0.099	-
A24	0.061	0.674	0.1	-	8.947

**Table 7:** Conditions of 72-bar truss structures.

Lumped Mass	*E*	ρ	*A*	Load	
				Case 1	Case 2
2270 kg	68,950 MPa	2767.99 kg/ $m^{3}$	−30 ${\mathrm{cm}}^{2}$ ≤ x ≤ 30 ${\mathrm{cm}}^{2}$	$F_{1x}$ = $F_{1y}$ = 22.25 kN, $F_{1z}$ = −22.25 kN	$F_{1z}$ = $F_{2z}$ = $F_{3z}$ = $F_{4z}$ = −22.25 kN

**Table 8:** The 72-bar TTO results (Unit: ${\mathrm{cm}}^{2}$ ).

Design Variable	Method I	Method II	Method III	Method IV	HSA
G1 (A1–A4)	4.880	5.655	7.985	4.776	6.574
G2 (A5–A12)	9.500	11.257	11.451	11.303	9.052
G3 (A13–A16)	5.651	-	-	-	-
G4 (A17–A18)	-	-	-	-	11.218
G5 (A19–A22)	15.002	7.500	6.573	9.264	18.603
G6 (A23–A30)	6.328	7.886	7.832	7.749	9.446
G7 (A31–A34)	-	-	-	2.873	-
G8 (A35–A36)	7.563	3.956	3.867	7.503	-
G9 (A37–A40)	11.549	15.074	15.485	9.864	12.434
G10 (A41–A48)	8.061	8.320	8.438	7.512	8.264
G11 (A49–A52)	-	-	-	-	-
G12 (A53–A54)	-	-	-	-	-
G13 (A55–A58)	14.581	15.032	14.063	18.199	14.989
G14 (A59–A66)	8.969	8.154	7.559	7.515	9.325
G15 (A67–A70)	-	-	-	-	-
G16 (A71–A72)	-	-	-	-	-

## Data Availability

Data are contained within the article.

## References

[1] Steane A. (1998). Quantum computing. Rep. Prog. Phys..

[2] Feynman R.P. (2018). Simulating physics with computers. Int. J. Theor. Phys..

[3] Deutsch D. (1985). Quantum theory, the Church–Turing principle and the universal quantum computer. Proc. R. Soc. Lond. A Math. Phys. Sci..

[4] Gharehchopogh F.S. (2023). Quantum-inspired metaheuristic algorithms: Comprehensive survey and classification. Artif. Intell. Rev..

[5] Horowitz M., Grumbling E., National Academies of Sciences, Engineering, and Medicine (2019). Quantum Computing: Progress and Prospects.

[6] Vedral V., Plenio M.B. (1998). Basics of quantum computation. PRogress Quantum Electron..

[7] McMahon D. (2007). Quantum Computing Explained.

[8] Choi M.S. (2022). A Quantum Computation Workbook.

[9] Hidary J.D., Hidary J.D. (2019). Quantum Computing: An Applied Approach.

[10] Kumar A., Bawa S. (2020). A comparative review of meta-heuristic approaches to optimize the SLA violation costs for dynamic execution of cloud services. Soft Comput..

[11] Agrawal P., Abutarboush H.F., Ganesh T., Mohamed A.W. (2021). Metaheuristic algorithms on feature selection: A survey of one decade of research (2009–2019). IEEE Access.

[12] Lee D., Kim J., Shon S., Lee S. (2023). An Advanced Crow Search Algorithm for Solving Global Optimization Problem. Appl. Sci..

[13] Han K.H., Kim J.H. (2000). Genetic quantum algorithm and its application to combinatorial optimization problem. Proceedings of the 2000 Congress on Evolutionary Computation. CEC00 (Cat. No. 00TH8512).

[14] Han K.H., Kim J.H. (2002). Quantum-inspired evolutionary algorithm for a class of combinatorial optimization. IEEE Trans. Evol. Comput..

[15] Yang S., Wang M. (2004). A quantum particle swarm optimization. Proceedings of the 2004 Congress on Evolutionary Computation (IEEE Cat. No. 04TH8753).

[16] Layeb A. (2013). A hybrid quantum inspired harmony search algorithm for 0–1 optimization problems. J. Comput. Appl. Math..

[17] Nezamabadi-Pour H. (2015). A quantum-inspired gravitational search algorithm for binary encoded optimization problems. Eng. Appl. Artif. Intell..

[18] Gao H., Zhang X., Du Y., Diao M. (2019). Quantum-inspired teaching-learning-based optimization for linear array pattern synthesis. Proceedings of the Communications, Signal Processing, and Systems: Proceedings of the 2017 International Conference on Communications, Signal Processing, and Systems.

[19] Hussain K., Mohd Salleh M.N., Cheng S., Shi Y. (2019). Metaheuristic research: A comprehensive survey. Artif. Intell. Rev..

[20] Hakemi S., Houshmand M., KheirKhah E., Hosseini S.A. (2022). A review of recent advances in quantum-inspired metaheuristics. Evol. Intell..

[21] Ross O.H.M. (2019). A review of quantum-inspired metaheuristics: Going from classical computers to real quantum computers. IEEE Access.

[22] Mohammed A.M., Elhefnawy N., El-Sherbiny M.M., Hadhoud M.M. (2012). Quantum crossover based quantum genetic algorithm for solving non-linear programming. Proceedings of the 2012 8th International Conference on Informatics and Systems (INFOS).

[23] Hakemi S., Houshmand M., Hosseini S.A., Zhou X. (2023). A Modified Quantum-Inspired Genetic Algorithm Using Lengthening Chromosome Size and an Adaptive Look-Up Table to Avoid Local Optima. Axioms.

[24] Yu L., Ren J., Zhang J. (2023). A Quantum-Based Beetle Swarm Optimization Algorithm for Numerical Optimization. Appl. Sci..

[25] Wong W., Ming C.I. (2019). A review on metaheuristic algorithms: Recent trends, benchmarking and applications. Proceedings of the 2019 7th International Conference on Smart Computing & Communications (ICSCC).

[26] Osaba E., Villar-Rodriguez E., Del Ser J., Nebro A.J., Molina D., LaTorre A., Suganthan P.N., Coello C.A.C., Herrera F. (2021). A tutorial on the design, experimentation and application of metaheuristic algorithms to real-world optimization problems. Swarm Evol. Comput..

[27] Alorf A. (2023). A survey of recently developed metaheuristics and their comparative analysis. Eng. Appl. Artif. Intell..

[28] Lee D., Shon S., Lee S., Ha J. (2023). Size and Topology Optimization of Truss Structures Using Quantum-Based HS Algorithm. Buildings.

[29] Lee S., Ha J., Shon S., Lee D. (2023). Weight Optimization of Discrete Truss Structures Using Quantum-Based HS Algorithm. Buildings.

[30] Geem Z.W., Kim J.H., Loganathan G.V. (2001). A new heuristic optimization algorithm: Harmony search. Simulation.

[31] Han K.H., Kim J.H. (2004). Quantum-inspired evolutionary algorithms with a new termination criterion, *H*_ϵ_ gate, and two-phase scheme. IEEE Trans. Evol. Comput..

[32] Savsani V.J., Tejani G.G., Patel V.K. (2016). Truss topology optimization with static and dynamic constraints using modified subpopulation teaching–learning-based optimization. Eng. Optim..

[33] Savsani V.J., Tejani G.G., Patel V.K., Savsani P. (2017). Modified meta-heuristics using random mutation for truss topology optimization with static and dynamic constraints. J. Comput. Des. Eng..

